# Volatile Fatty Acids Effective as Antibacterial Agents against Three Enteric Bacteria during Mesophilic Anaerobic Incubation

**DOI:** 10.3390/molecules29091908

**Published:** 2024-04-23

**Authors:** Saanu Victoria Otite, Alfonso José Lag-Brotons, Lawrence I. Ezemonye, Alastair D. Martin, Roger W. Pickup, Kirk T. Semple

**Affiliations:** 1 Lancaster Environment Centre, Library Avenue, Lancaster University, Lancaster LA1 4YQ, UK; s.obatusin@lancaster.ac.uk; 2 Engineering Department, Gillow Avenue, Lancaster University, Lancaster LA1 4YW, UK; 3 Centre for Global Eco-Innovation Nigeria, University of Benin, Benin City PMB 300313, Nigeria; 4 Vice Chancellor’s Office, Igbinedion University Okada, Benin City PMB 0006, Nigeria; 5 Division of Biomedical and Life Sciences, Furness Building, Lancaster University, Lancaster LA1 4YG, UK

**Keywords:** organic acids, bacterial elimination, minimum bactericidal concentration, *Enterococcus faecalis* resistance, acidogenic anaerobic digestion

## Abstract

The antibacterial effects of a selection of volatile fatty acids (acetic, propionic, butyric, valeric, and caproic acids) relevant to anaerobic digestion were investigated at 1, 2 and 4 g/L. The antibacterial effects were characterised by the dynamics of *Enterococcus faecalis* NCTC 00775, *Escherichia coli* JCM 1649 and *Klebsiella pneumoniae* A17. Mesophilic anaerobic incubation to determine the minimum bactericidal concentration (MBC) and median lethal concentration of the VFAs was carried out in Luria Bertani broth at 37 °C for 48 h. Samples collected at times 0, 3, 6, 24 and 48 h were used to monitor bacterial kinetics and pH. VFAs at 4 g/L demonstrated the highest bactericidal effect (*p* < 0.05), while 1 g/L supported bacterial growth. The VFA cocktail was the most effective, while propionic acid was the least effective. *Enterococcus faecalis* NCTC 00775 was the most resistant strain with the VFAs MBC of 4 g/L, while *Klebsiella pneumoniae* A17 was the least resistant with the VFAs MBC of 2 g/L. Allowing a 48 h incubation period led to more log decline in the bacterial numbers compared to earlier times. The VFA cocktail, valeric, and caproic acids at 4 g/L achieved elimination of the three bacteria strains, with over 7 log_10_ decrease within 48 h.

## 1. Introduction

An understanding of the biochemical processes and intermediate substances produced during anaerobic digestion (AD) of organic wastes can aid in optimising the technology for disinfecting the products. Several factors have been reported to be responsible for pathogen removal during AD, but these depend on the configuration being used [1]. Temperature is the most important sanitising factor in thermophilic AD [2], while factors such as residence time [3,4], pH and presence of inhibitory substances such as volatile fatty acids [5,6,7] and ammonia have been attributed to pathogen removal during mesophilic AD [8]. Mesophilic (25–40 °C) AD is the commonest type of AD used due to its ease of operation and low energy cost. Despite these benefits, it cannot be used to produce class A effluent (no detectable pathogen levels) due to its inability to achieve a high log decrease in pathogen numbers. Consequently, there is a need for further understanding of the critical factors involved in pathogen inactivation during mesophilic AD, especially how the biochemical processes and conditions can enhance its sanitary effect. Some studies have shown that the acidogenic stage of AD is its sanitary phase due to the accumulation of volatile fatty acids [1,3,6].

Volatile fatty acids (VFAs) are short-chain organic acids which are made up of one to six carbon moieties. They are produced as intermediates of fermentation processes [9]. Acetic, propionic, butyric, valeric, isovaleric, and caproic acids are the VFAs that are produced in varying amounts during AD, depending on the substrate type [10,11,12]. VFAs have been reported to possess antimicrobial properties due to the ability of their lipophilic undissociated forms to cross the bacterial cell membrane and alter cytoplasmic electrochemical gradient [13,14], thus leading to cell death. This sanitary effect is enhanced under low pH (less than the pKa of VFAs, which is approximately 4.5) since acidity favours the non-dissociation of VFAs at their dissociation constant (pKa) [13]. Thus, volatile fatty acids have a non-thermal and cost-effective advantage as an antibacterial agent during mesophilic AD.

*Enterococcus faecalis, Escherichia coli* and *Klebsiella pneumoniae* are enteric facultative non-sporing bacteria found in the gastrointestinal tracts of humans and animals and in faecal wastes [15,16,17]. These organisms can become potentially pathogenic to humans when they find their way into soil and water environments through unsafe handling practices of human excrement and animal manure, such as the use of untreated faecal wastes as fertiliser, as these bacteria are transmissible through the faecal–oral route. The virulent strains could cause gastroenteritis or more fatal illnesses such as hemolytic uremic syndrome and pneumonia [18,19,20]. Furthermore, they have all been implicated as common causes of bacteremia in immunosuppressed people or patients undergoing invasive surgical procedures [21]. These bacteria may also possess varying degrees of resistance to antimicrobial treatments and environmental conditions [8,15].

Organic acids have mostly been used by the food industry and, therefore, tested against foodborne pathogens during food and feed production [21,22,23,24,25,26,27]. Previous studies on the effects of different doses of organic acids on meat spoilage bacteria [28,29], *Clostridium perfringens* and *C. difficile* [30,31], *Staphylococcus aureus* and *Escherichia coli* [32], enteropathogenic bacteria [14], demonstrated that they have the potential to act as antimicrobial agents. However, their antibacterial efficacy against enteric pathogens in the environment has not been fully explored, particularly in biological waste treatment plants where VFAs are produced as intermediates of such processes. Most environmental studies on the antibacterial effects of organic acids considered VFA activities in vivo during AD [33,34]; however, other digester conditions may contribute to or negate the effects of these VFAs on targeted species. Other reports focused on in vitro studies using mainly medium to long-chain organic acids and their derivatives [24,35] or reported mainly the minimum inhibitory concentrations of the organic acids [35,36]. However, there has been no comprehensive report on the in vitro effects of several VFA types, which are likely to be produced during the acidogenic phase of AD, on pathogen kinetics. This study therefore aimed to (i) assess the in vitro antibacterial effects of C_2_ to C_6_ VFA types and concentrations on a typed strain of *Enterococcus faecalis* and environmental strains of *Escherichia coli* and *Klebsiella pneumoniae* under mesophilic anaerobic incubation, (ii) determine the kinetics of the named enteric bacteria over a 48-h period, and (iii) determine the median lethal concentrations and minimum bactericidal concentrations of the VFAs on the named bacteria. Results from this study could enhance the design and optimisation of mesophilic acidogenic anaerobic digestion.

## 2. Results

### 2.1. Effect of VFA Concentrations on Bacteria Kinetics

The antibacterial effect of all the tested volatile fatty acids was observed to be concentration-dependent, with 4 g/L being the most effective and 1 g/L the least effective. The percentage decreases in the counts for the three bacterial strains over time for the three VFA concentrations are shown in Table 1, Table 2 and Table 3.

#### VFAs at 1, 2 and 4 g/L

The VFAs concentration at 1 g/L did not achieve a significant decrease (*p* < 0.05) in counts of all three bacterial strains. Although some initial decrease was observed with *E. faecalis* NCTC 00775 within the first 6 h with the lower molecular weight VFAs and the VFA cocktail, the strain recovered from this effect and had an increase in counts at the end of the 48 h incubation period (Table 1). An exception to this recovery was acetic acid, which achieved 76.71% (less than 1 log) at the end of the incubation period. None of the VFAs at 1 g/L achieved a decrease in *E. coli* JCM 1649 numbers; rather, there was an increase in cell numbers across all time points (Table 2). *K. pneumoniae* A17 was also initially susceptible to most of the VFAs at 1 g/L except acetic and caproic acids, but at the end of the incubation period, acetic, butyric, and valeric acids, as well as the VFA cocktail, achieved 97.71%, 43.54%, 86.08% and 64.92% decrease in *K. pneumoniae* A17 CFUs, respectively (Table 3). Thus, when compared to growth in the LB broth control, the antibacterial efficacy of the VFAs against the tested strains at 1 g/L was generally statistically insignificant (*p* < 0.05) (Table S1).

At 2 g/L, all the VFAs resulted in varying decreases in log CFU/mL counts of *E. faecalis*, but none attained up to one log kill (90%) (Table 1). However, unlike the concentration at 1 g/L, there was no increase in CFUs, apart from valeric acid, where there was an initial increase after 3 h of incubation, followed by a subsequent decline. At 2 g/L concentration, propionic, butyric, and caproic acids achieved more than a 90% decrease, while acetic acid and the VFA cocktail achieved more than 2 log (99%) decrease in *E. coli* counts at the end of the incubation period (Table 2). Furthermore, at 2 g/L, all the VFAs achieved between 90% and 100% decrease in *K. pneumoniae* numbers, with acetic acid being the least effective (Table 3).

At 4 g/L, the antibacterial effect of VFAs increased with an increase in carbon chain length. The VFA cocktail achieved elimination of *E. faecalis* below the detection level after 6 h; caproic and valeric acids after 24 h; while butyric, acetic and propionic acids achieved 99.96%, 99.96%, 99.71% kill, respectively, after 48 h (Table 1). The VFA cocktail achieved complete elimination of *E. coli* in less than 3 h, caproic and valeric acids after 3 h, acetic acid after 6 h, as well as butyric and propionic acids after 24 h (Table 2). The VFA cocktail, valeric, and butyric acids achieved complete elimination of *K. pneumoniae* in less than 3 h, caproic acid after 6 h, and propionic acid after 24 h, while acetic acid achieved only 99.90% decrease in CFUs (Table 3). There was a more toxic effect and death with prolonged contact time between bacterial strains and the VFAs.

### 2.2. Effect of VFA Types on Bacterial Strains

**Figure 1:**
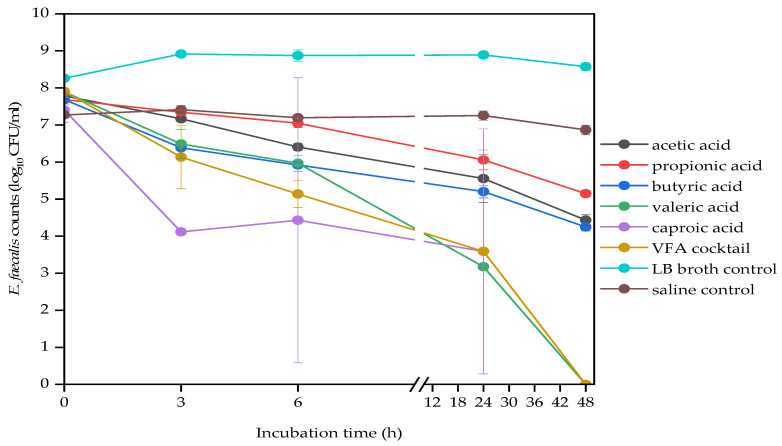
Effect of VFA types (C2 to C6) at 4 g/L on *E. faecalis* counts during a 48 h mesophilic anaerobic incubation in LB broth. LB broth and saline are controls accounting for natural dynamics in the absence of VFAs and nutrients, respectively. Data points represent the mean and standard deviation of 3 readings.

**Figure 2:**
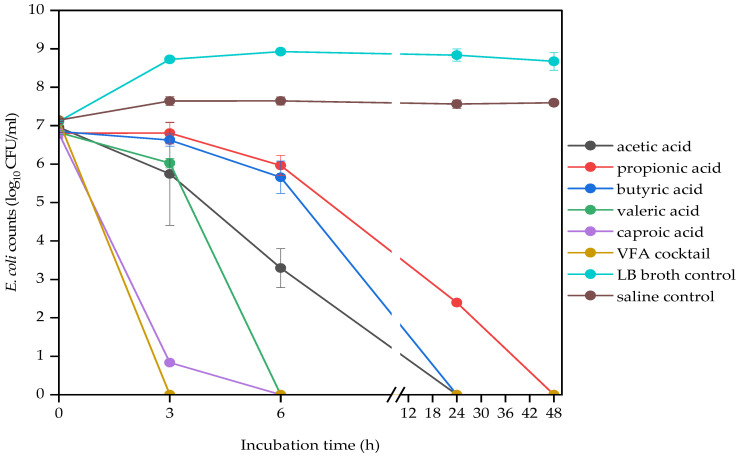
Effect of VFA types (C2 to C6) at 4 g/L on *E. coli* counts during a 48 h mesophilic anaerobic incubation in LB broth. LB broth and saline are controls accounting for natural dynamics in the absence of VFAs and nutrients, respectively. Data points represent the mean and standard deviation of 3 readings.

**Figure 3:**
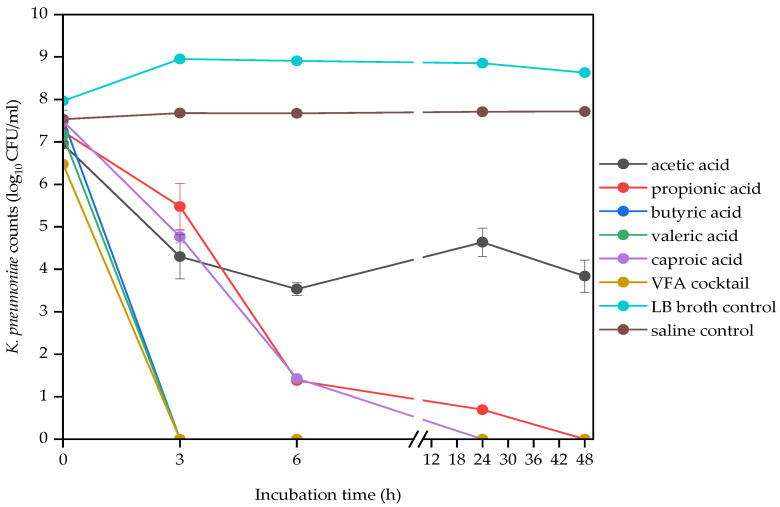
Effect of VFA types (C2 to C6) at 4 g/L on *K. pneumoniae* counts during a 48 h mesophilic anaerobic incubation in LB broth. LB broth and saline are controls accounting for natural dynamics in the absence of VFAs and nutrients, respectively. Data points represent the mean and standard deviation of 3 readings.

The antibacterial efficacy of VFAs varied according to the VFA type. The effect of VFA type on bacterial strains was shown using the data from 4 g/L concentration (Figure 1, Figure 2 and Figure 3), since the most significant difference was observed at this concentration. Generally, the VFA cocktail and the higher molecular weight VFAs, caproic (C6) and valeric acids (C5), were the most toxic ones. While propionic (C3) acid was the least toxic against the three bacterial strains. The lower molecular weight VFAs, propionic, acetic and butyric acids, were the least effective against *E. faecalis*, demonstrated by significant log CFUs left after the 48 h incubation period (Figure 1). The VFA cocktail was the most effective, while propionic acid was the least effective VFA against *E. coli* (Figure 2). The VFA cocktail, valeric and butyric acids were the most effective, while acetic acid was the least effective VFA against *K. pneumoniae* (Figure 3). Table 4 also shows an overview of the antimicrobial activities of the VFAs on the three bacterial strains.

### 2.3. Effect of Bacterial Types on the Antibacterial Efficacy of VFAs

**Figure 4:**
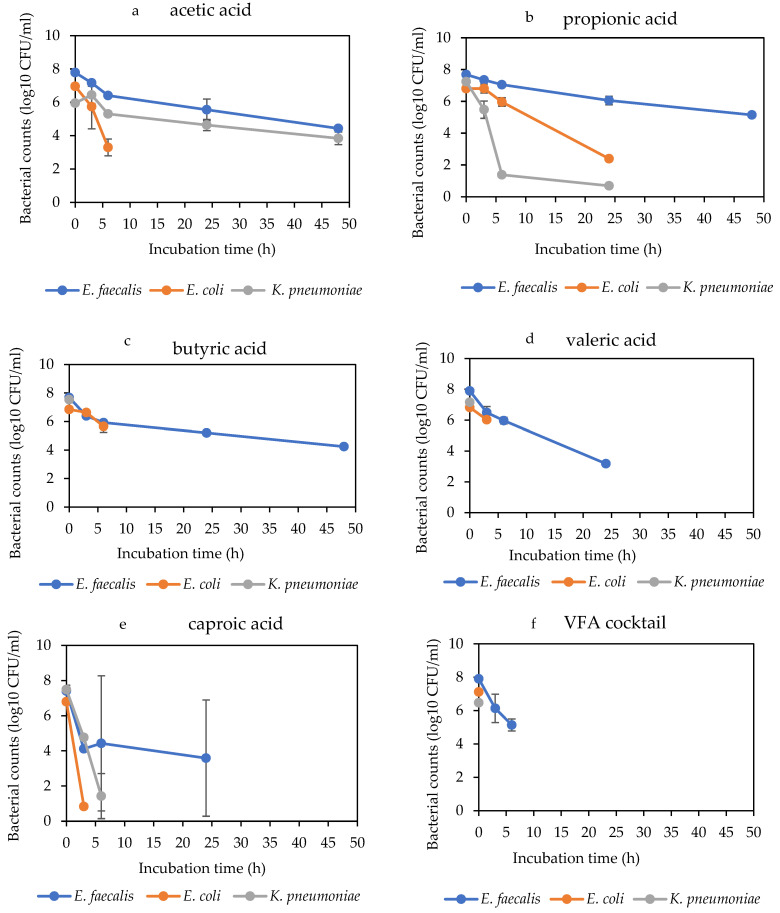
Comparison of the death kinetics of *E. faecalis*, *E. coli* and *K. pneumoniae* during a 48 h mesophilic anaerobic incubation at 4 g/L of VFAs. Graphs (**a**–**f**) are the VFA types.

The antibacterial efficacy of VFAs varied with the bacterial type. *E. faecalis* was the most resistant strain. This was demonstrated by its survival in significant numbers in the presence of more VFA types, specifically the C2 to C4 VFAs, acetic, propionic, and butyric acids at 4 g/L even after 48 h (Figure 4), and also by showing relatively high MBC of 4 g/L for all the VFAs (Table 5). Furthermore, the VFAs at 2 g/L did not achieve up to 1 log decrease in CFUs with *E. faecalis*. Although *E. coli* and *K. pneumoniae* demonstrated similar levels of susceptibility to the VFAs, *K. pneumoniae* was the most susceptible of the three bacterial species, being eliminated by more VFAs within a shorter time and being susceptible to the VFAs mostly at MBC of 2 g/L compared to *E. coli* (Table 6 and Table 7).

### 2.4. LC_50_, MBC, and Comparative Toxicity of VFAs

The LC_50_ and MBCs of VFAs against *E. faecalis*, *E. coli* and *K. pneumoniae* over time are shown in Table 5, Table 6 and Table 7, respectively. The LC_50_ of all VFAs decreased with an increase in time for *E. coli*. While for *E. faecalis*, this pattern was observed with only acetic, propionic and butyric acids; there were some fluctuations with the VFA cocktail, as well as valeric and caproic acids. For *K. pneumoniae*, there were fluctuations in the LC_50_ over time. At the end of the 48 h incubation period, acetic acid had the lowest LC_50_ for *E. faecalis* and *K. pneumoniae*, at 0.77 and 0.08 g/L, respectively, while the VFA cocktail had the lowest LC_50_ for *E. coli* at 1.85 g/L. Butyric and propionic acids had the highest LC_50_ for *E. faecalis* at 2.13 and 2.12 g/L, respectively; propionic and valeric acid for *E. coli,* both at 2.06 g/L; and caproic acid for *K. pneumoniae at* 1.85 g/L. The MBC for all the VFAs with *E. faecalis* was 4 g/L, and this was mostly after 48 h of incubation; the MBC was beyond the tested concentration of 4 g/L at the other time points. For *E. coli*, the MBC of the VFAs was also 4 g/L, but this was at varying time points, while the VFA cocktail had an MBC of 2 g/L. The MBC for VFAs with *K. pneumoniae* was 2 g/L at 48 h, except for acetic acid, which had an MBC of 4 g/L at 48 h. The MBC at the other time points was 4 g/L, with a few beyond the detection limit of 4 g/L.

### 2.5. pH Dynamics during Bacterial Incubation

**Figure 5:**
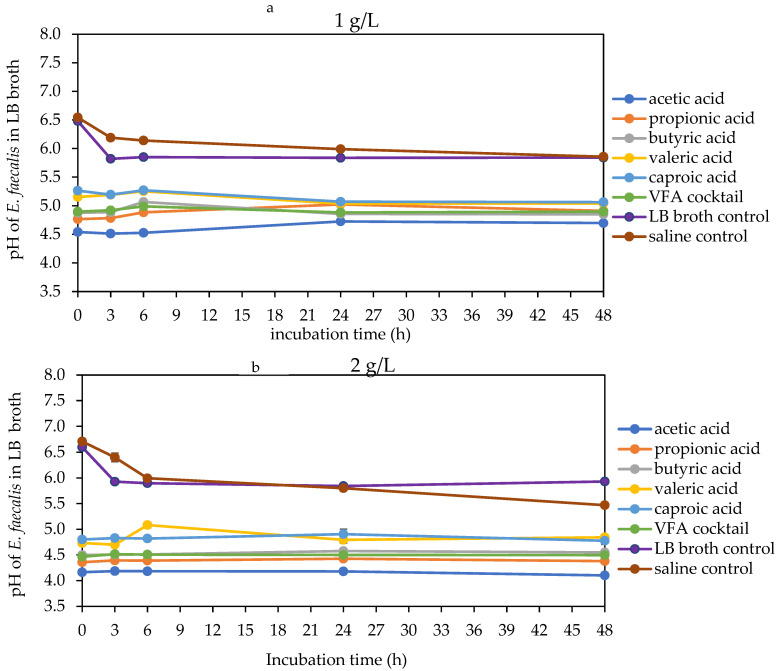

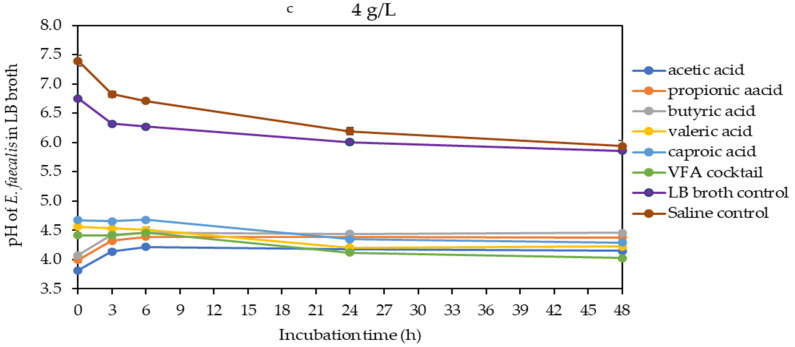
pH of LB broth with different VFA types during *E. faecalis* 48 h mesophilic anaerobic incubation. LB broth and saline are controls accounting for natural dynamics in the absence of VFAs and nutrients, respectively. Graphs (**a**–**c**) are the VFA concentrations (1, 2 and 4 g/L).

**Figure 6:**
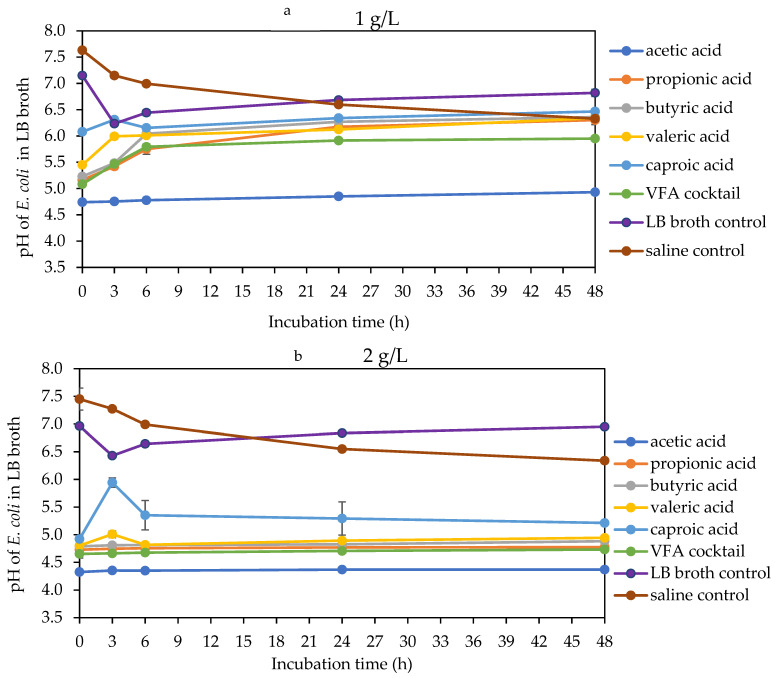

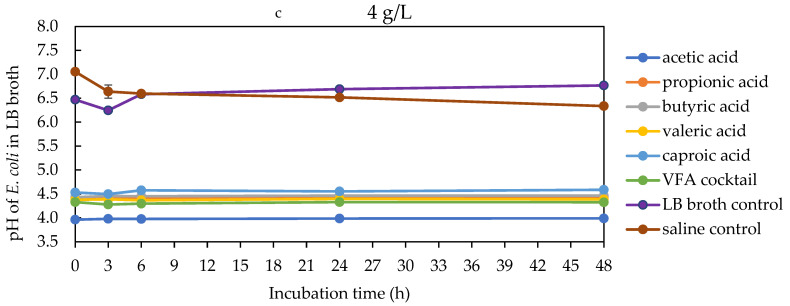
pH of LB broth with different VFA types during *E. coli* 48 h mesophilic anaerobic incubation. LB broth and saline are controls accounting for natural dynamics in the absence of VFAs and nutrients, respectively. Graphs (**a**–**c**) are the VFA concentrations (1, 2 and 4 g/L).

**Figure 7:**
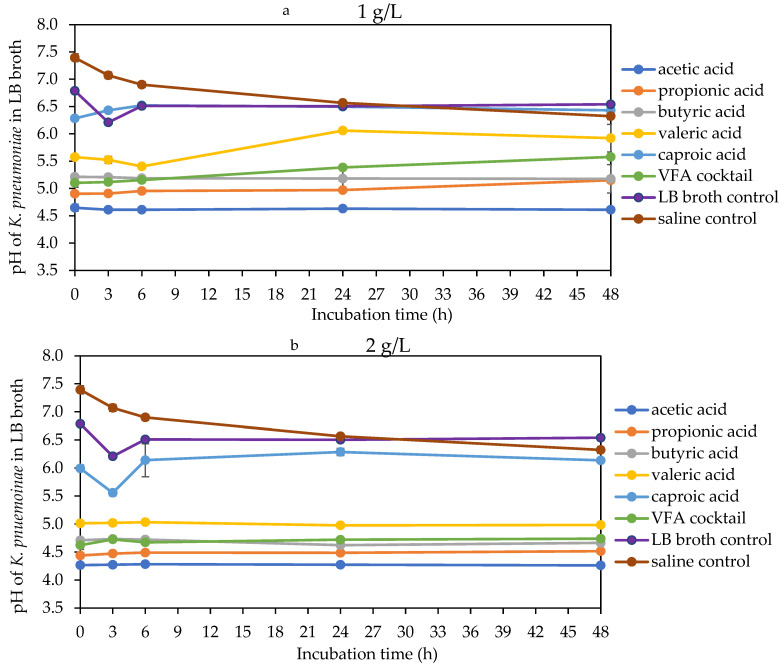

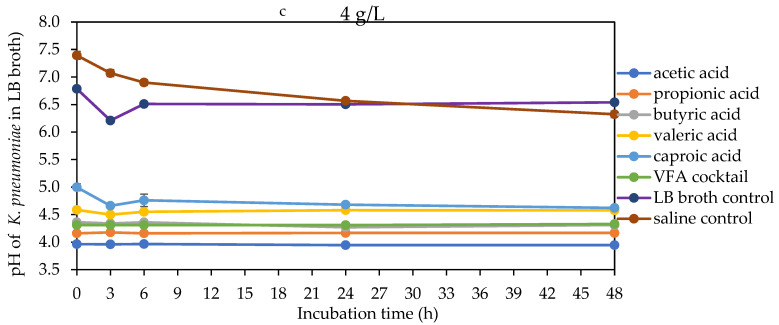
pH of LB broth with different VFA types during *K. pneumoniae* 48 h mesophilic anaerobic incubation. LB broth and saline are controls accounting for natural dynamics in the absence of VFAs and nutrients, respectively. Graphs (**a**–**c**) are the VFA concentrations (1, 2 and 4 g/L).

The acidity of the bacterial culture broth increased with an increase in VFA concentration and decreased with an increase in VFA molecular weight. Hence, acetic acid at 4 g/L had the lowest pH, while caproic acid at 1 g/L had the highest pH across all bacterial broth (Figure 5, Figure 6 and Figure 7). At 1 g/L, there was no difference in pH for all the VFAs with *E. faecalis*, while for *E. coli*, there was a marked increase in pH with all VFAs except acetic acid, whereas, for *K. pneumoniae*, there was only a significant increase with caproic and valeric acids (Figure 5a, Figure 6a and Figure 7a). At 2 g/L, there were changes in the pH of valeric acid for *E. faecalis* and caproic acid for *E. coli* and *K. pneumoniae* (Figure 5b, Figure 6b and Figure 7b). At 4 g/L, there was no marked change in pH during the incubation period. At this concentration, the pH ranged from about 3.9 with acetic acid to about 4.6 with caproic acid across all the bacterial strains (Figure 5c, Figure 6c and Figure 7c). There was an initial decrease in the pH of the saline and LB broth controls within the first 3 h of incubation. This was followed by a more stable pH through the rest of the incubation period for *E. faecalis*. On the other hand, this initial decrease in pH was followed by an increase in pH within the third and sixth hours for both *E. coli* and *K. pneumoniae*. Most of the changes in the pH of the bacteria broth culture occurred within the first 6 h of incubation, followed by stability afterwards.

## 3. Discussion

The *E. faecalis* NCTC 00775, *E. coli* JCM 1649 and *K. pneumoniae* A17 strains, which were used in this study, were confirmed to be multidrug-resistant based on preparatory antibiotic susceptibility testing prior to this investigation (Table S2). These organisms are commonly present in organic wastes, especially those of human and animal origin, and may pose a public health risk if they remain in untreated or improperly treated materials which are recycled into the environment. This study opened up a further understanding of the utilisation of AD intermediates, such as volatile fatty acids, as antibacterial agents to decrease pathogen numbers.

### 3.1. The Antibacterial Effect of VFAs as a Function of Concentration and pH

The toxicity of VFAs increased with an increase in concentration. This is expected with most toxic substances, which achieve an increase in lethal effect as the concentration increases until the optimum lethal concentration is reached [1,37]). Also, short-chain fatty acids (SCFA) enter the cell cytoplasm through passive diffusion. Thus, the more concentrated they are in the growth medium, the more available they are to move into the cell; hence, the VFAs at 4 g/L were more effective against the tested bacterial strains. This agrees with Kim and Lee [17], who reported the dose-dependent nature of organic acids and also the complete inactivation of *E. coli* after 48 h of incubation when treated with 4 g/L of acetate, propionate and butyrate. Furthermore, the toxicity of VFAs is correlated with the pH of the growth medium (Figure S1), as also observed by Peh et al. [38], because the ratio of the ionised form of VFA to the unionised form is influenced by pH. The pKa of all the tested VFAs (approximately 4.8) lies within the acidic pH, making it more favourable to pathogen inactivation conditions [39]. The pH of the medium was lowest at VFA concentrations of 4 g/L, indicating high acidity, and there was no change in the pH of the growth medium during the incubation period at this concentration, indicating that only a small proportion of the VFA was involved in metabolism, as also observed by Fang et al. [24]. The antibacterial mechanisms of organic acids against bacteria include cell lysis due to osmotic shock, imbalance in the electrochemical gradient of the cell, or acidification of cell cytoplasm with attendant disruption of DNA and protein synthesis [40]. Thus, the susceptibility of the tested bacterial strains to VFAs at 4 g/L is potentially due to the inability of the strains to adjust their intracellular and extracellular pH in the presence of such high amounts of VFAs, which led to the cytoplasmic accumulation of acid anions at lethal doses due to inward diffusion of undissociated VFAs. On the other hand, the VFAs concentration at 1 g/L did not achieve a significant bacterial kill, likely because their relatively low presence in the bacterial environment resulted in cytoplasmic concentration, which is neither able to cause permanent disruption of the cell electrochemical gradient nor lead to osmotic shock. This agrees with Ouattara et al. [28], who also found bacterial resistance to organic acids at concentrations lower than 2.5 g/L. The decrease in counts observed with 1 g/L of acetic acid on *E. faecalis* and *K. pneumoniae* may be because low molecular weight VFAs are hydrophilic and can pass through the cell membrane by diffusing through the porin proteins in small amounts, despite the VFAs being present in low concentration in the surrounding medium. It could also be attributed to extracellular pH shock due to the sudden drop in pH of the medium from about 6.5 to 4.5. This agrees with the study of Gomez-garcia [14], who observed that formic and propionic acids, which are low molecular weight VFAs, showed high toxicity to bacteria. In addition, the kill achieved with *K. pneumoniae* by acetic, butyric, VFA cocktail and valeric acid at this concentration could indicate the susceptibility of this strain to VFAs [41]. The resistance of *E. coli* to all the VFAs at 1 g/L may be due to its ability to express acid-shock proteins [42], develop acid tolerance response (ATR) [43] as well as decrease its intracellular pH close to that of the surroundings, and adjust its cell electrochemical gradient. This ability to adjust the pH of the growth medium was strongly correlated with the recovery and survival of tested strains during the incubation period. This was demonstrated by the marked increase in pH of the *E. coli* growth medium at 1 g/L of VFAs. Slabbert et al. [44] also observed that *E. coli* induced acid tolerance response (ATR) over time and was able to grow at suboptimal pH levels. The concentration of VFAs at 1 g/L led to an increase in counts of bacterial species compared to the initial concentration at time zero. This agrees with Nair et al. [45], who also observed an increase in *E. coli* counts when sub-lethal concentrations of caprylic acid and monocaprylin were used, and Salsali et al. [30], who observed an increase in the counts of *Clostridium perfringens* when 0.75 g/L of VFAs was present during AD. Bacteria can use organic acids as carbon and energy sources; hence, the increase in CFUs observed at this concentration could be due to the ability of the organisms to carry out normal cellular activities and multiplication, compared to the concentration at 2 g/L where organisms were unable to grow and multiply. The significance of this while running AD is that sub-lethal doses of VFAs could lead to the growth of pathogens, which could result in high pathogen loading in digestate.

### 3.2. Effect of Carbon Chain Length on VFAs Toxicity

The antibacterial effect of VFAs increased with an increase in the carbon chain length and, hence, the molecular weight, particularly at higher VFA concentrations [1]. Thus, caproic and valeric acids were the most effective VFAs against all the tested strains when tested separately. Kovanda et al. [35] also reported that valeric acid was effective against the tested Gram-negative and Gram-positive bacteria. This could be because the polar nature of high molecular weight VFAs causes interference with the bacterial cell membrane and alters the fluidity [46]. Although the VFA cocktail demonstrated the overall highest antibacterial activity against all tested bacterial strains, this could indicate a synergistic lethal effect of the VFA combination against target organisms, as also previously reported [23,25,38,47]. Although the general trend of increasing toxicity with an increase in chain length was observed in this study, a deviation was noted with acetic acid, which demonstrated high toxicity against *E. coli* despite being a C_2_ acid. Al-Rousan et al. [47] also reported the toxic effect of acetic acid against *E. coli* at 4 g/L. This could be because of the inhibitory effect of the low pH (3.9 at 4 g/L) of this VFA [48]. Hence, there seems to be a synergistic effect between the inhibitory effect of acetic acid and its low pH, as also reported by Breidt et al. [49]. Furthermore, Gomez-garcia et al. [14] also observed that formic acid, which is also a low molecular weight VFA (C_1_) with low pH, showed higher toxicity to *Escherichia coli* and *Salmonella* spp. Although propionic acid was comparatively the least effective VFA against all tested strains in this study, it is a potent antibacterial agent [17,50]. It achieved a significant reduction (>99%) in bacterial log CFUs at 4 g/L within the 48 h incubation period.

### 3.3. Varied Susceptibility of Bacterial Strains to VFAs

*E. faecalis* was the most resistant species, particularly with the lower molecular weight VFAs: acetic, propionic, and butyric acids. *E. faecalis* is known to be resistant to a wide range of temperature, pH and other stressful environmental conditions [51,52,53]. This agrees with Kovanda et al. [35], who also reported the resistance of *E. faecalis* to butyric acid with a MIC of 2 g/L. Hence, the ability of high molecular weight hydrophobic VFAs to alter and penetrate the cell wall peptidoglycan and reach the cytoplasm could be responsible for their lethal effect against *E. faecalis*. In this study, *K. pneumoniae* and *E. coli* were more susceptible to the VFAs; this could be because they are Gram-negative bacteria and possess a thin layer of the cell wall with limited selective inhibition of the inflow of toxic agents. Mapipa et al. [54] reported the sensitivity of *K. pneumoniae* to antimicrobial agents, and Adamczak et al. [36] also reported that Gram-negative bacteria were comparatively more susceptible to organic acids than Gram-positive bacteria. Despite the relative susceptibility of *K. pneumoniae* to the tested VFAs, it was more resistant to acetic acid than the other two species. This could be because acetic acid is among the products of metabolism of *K. pneumoniae* [55] and has developed resistance to it at the tested concentrations due to the expression of acid shock proteins. This observation disagrees with Krusong et al. [56], who reported that acetic acid completely inhibited the growth of *K. pneumoniae*. This disparity could be due to the use of 2% concentration by the authors, compared to 0.4% acetic acid used in this study.

### 3.4. LC_50_ and MBC as Indicators of VFA Toxicity

The lower the LC_50_ of a toxic substance, the more potent it is expected to be; this means a lower concentration of that substance can kill 50% of the target population [57]. Although acetic acid had the lowest LC_50_ for *E. faecalis* and *K. pneumoniae*, it did not have the lowest MBC for them within the shortest time. MBC, being the lowest concentration that will achieve a significant log decrease, that is, greater than 3log_10_ [58], could be a more relevant indicator of the sanitary potency of VFAs in AD. The VFA cocktail and caproic acid had an MBC of 4 g/L within the shortest time and thus were the most effective against the three tested bacterial strains. Pelyuntha and Vongkamjan [59] also reported a higher MBC of 5 g/L, 7.5 g/L and 7.5 g/L for acetic, propionic and butyric acids, respectively, against *Salmonella* Enteritidis. In this study, we observed that LC_50_ may not be a good indicator of the antibacterial potencies of VFAs, especially in biotreatment processes where temporary inhibition and re-growth of bacterial species can occur, as demonstrated by the haphazard pattern of LC_50_ in *E. faecalis* and *K. pneumoniae*. A concentration which demonstrates a significant amount of kill (above 99.9%) could be more promising.

### 3.5. Influence of Incubation Time on VFAs Toxicity

The time-kill curve is an important tool for determining microbial dynamics in antimicrobial studies [60]. In this study, it was observed that more log kill was achieved between 24 and 48 h of incubation. This demonstrates the importance of contact time in bacteria inactivation studies [7]. Especially for the low molecular weight VFAs, which did not achieve a significant decrease before 24 h. This time-kill effect was also reported by Fan et al. [61], who assessed the time-dependent effect of lactobionic acid on *Vibrio parahaemolyticus*. This time-kill information is useful in designing AD for sanitary purposes, and hence, monitoring the acidogenic phase of AD within the first 24 to 48 h is important. The VFA cocktail, which is the expected combination in most natural anaerobic bioprocesses, was the most effective; this confirms that acidogenic AD is a promising method of sanitising organic wastes.

## 4. Materials and Methods

### 4.1. Bacterial Strains and Culture Media

*Enterococcus faecalis* NCTC 00775 was procured from Public Health England-National Collection of Type Cultures. *Escherichia coli* JCM 1649 and *Klebsiella pneumoniae* A17 were previously isolated and identified using Sanger sequencing from anaerobic digesters treating simulated food waste and animal manure. All strains were maintained as glycerol stock cultures at −30 °C. Luria-Bertani (LB) broth (Sigma Aldrich, Gillingham, Dorset, UK) was used for bacteria broth culture incubation. LB broth with agar (Sigma Aldrich, UK) was used for bacterial enumeration. Brilliance *E. coli* coliform selective medium (Oxoid, Basingstoke, Hampshire, UK) was used to recover the *E. coli* and *K. pneumoniae* colonies, while Slanetz and Bartley medium (Oxoid, UK) was used for *E. faecalis* recovery from the glycerol stocks. All culture media were prepared according to the manufacturer’s instructions. The antibiotic resistance patterns of the strains were pre-determined using the disc diffusion method following breakpoint values by the Clinical and Laboratory Standard Institute [62]. This was performed to establish the resistant nature of target strains to known common antibiotics. The antibiogram of the strains used is shown in Table S2 of the Supplementary Materials.

### 4.2. Volatile Fatty Acids (VFAs) Preparation

Five reagent grade VFAs: acetic (100%, s 1.05 g/cm), propionic (99.5%, s 0.99 g/cm), butyric (99%, s 1.14 g/cm), valeric (99%, s 0.94 g/cm) and caproic acids (96%, s 0.93 g/cm) in solution were procured from Sigma Aldrich, UK. A VFA cocktail was also prepared by mixing an equal volume of the five aforementioned VFAs. The VFAs were sterilised using a 0.22 µm filter and diluted with sterile deionised water to obtain three concentrations of 10%, 20% and 40% *v*/*v* VFA. These dilutions were used as the working stock.

### 4.3. Preparation of Bacterial Broth Cultures

Broth cultures of *E. coli* JCM 1649, *K. pneumoniae* A17 and *E. faecalis* NCTC 00775 were prepared in sterile 15 mL culture tubes containing 5 mL of freshly prepared LB broth. Culture tubes were incubated at 37 °C for 12–18 h (overnight incubation). Bacterial cell concentration was standardised by absorbance reading with a spectrophotometer at an optical density (OD) of 600 nm and a plate count to obtain a stock suspension of 10^7^ CFU/mL. For each experimental set-up, 5 mL of overnight bacterial stock culture of each strain was inoculated into 500 mL of sterile LB broth and incubated for 1 h to enable the strains to pass the lag phase of growth and acclimatise in the fresh medium. This resulted in a starting bacterial concentration of between 10^7^ and 10^8^ CFU/mL.

### 4.4. Experimental Design and Set Up

The experimental design was full factorial, comprising: (a) VFA type (*n* = 6; acetic, propionic, butyric, valeric, caproic and VFA cocktail); (b) VFA concentration (*n* = 3; 1, 2 and 4 g/L); (c) bacterial species (*n* = 3; *E. faecalis* NCTC 00775, *E. coli* JCM 1649 and *K. pneumoniae* A17); and (d) time (*n* = 5; 0, 3, 6, 24 and 48 h). Additionally, controls without VFA, made up of bacterial culture in saline and in LB broth, were included to assess the natural decline of bacteria due to senescence and optimum bacterial growth in the presence of nutrients, respectively. The experiment was carried out in 15 mL culture tubes. Then, 9.9 mL of the acclimated LB broth culture of each strain was dispensed separately into each well-labelled 15 mL tube. To these, 0.1 mL VFA of appropriate concentration (10%, 20% and 40%) was added to give final VFA concentrations of 0.1%, 0.2% and 0.4% (1 g/L, 2 g/L and 4 g/L), respectively, in the broth culture. These concentrations were chosen as they have been reported to be the range of VFAs produced during mesophilic AD [63,64]. The tubes were capped loosely to allow for gaseous exchange and anaerobiosis and arranged into anaerobic chambers (ThermoFisher Scientific AnaeroGen^TM^ 2.5 L, Waltham, MA, USA) with a gas generating kit (ThermoFisher Scientific AN0025A) and resazurin anaerobic indicator (ThermoFisher Scientific BR0055B). The anaerobic chambers containing the experimental tubes were incubated at 37 °C with shaking at 180 rpm for 48 h.

### 4.5. Sampling

Samples were collected at 0, 3, 6, 24 and 48 h destructively. At each time point, 0.1 mL of sample was collected for bacterial enumeration, while the remaining content of the tube was used for pH measurement. These analyses were performed immediately after sampling. Time 0 sample was collected immediately after adding the VFA into the culture broth and homogenised by swirling for uniform mixing. For the other time points, an individual anaerobic chamber was brought out of the incubator at the stipulated time.

#### 4.5.1. Bacterial Enumeration

The samples collected at each time point were serially diluted by a factor of 10 using 9.9 mL of phosphate-buffered saline. Then, 0.1 mL of selected dilutions were plated on LB agar (Oxoid, UK) and incubated at 37 °C for 24 h. Colony forming units (CFUs) per ml of sample were recorded as an average of log_10_ CFU/mL from three replicates.

#### 4.5.2. pH Measurement

The pH of samples at each time point was determined using a pH electrode (Mettler Toledo, Columbus, OH, USA) by dipping the electrode into the tubes containing approximately 9.9 mL of broth. The average pH readings of three replicates were recorded.

### 4.6. Determination of Median Concentration and Minimum Bactericidal Concentration

The median concentration (LC_50_), which was the concentration of VFAs that killed 50% of the bacterial strains at a given time, was determined using the Probit method [65]. Briefly, the Probit values of the bacterial death percentage were plotted against a log of VFA concentrations. The estimated LC_50_ was calculated using the regression of the dose-response graph. The minimum bactericidal concentration (MBC) of the VFAs was determined by the concentration of VFAs that achieved 3 log elimination (99.9%) [62] of the bacteria cells at a given time point when the bacteria broth was cultured on a solid agar plate.

### 4.7. Statistical Analysis

VFA type (“type”) and concentration (“conc”) were assessed for descriptive and inferential statistics. Time (“t”) and bacterial species (“sp”) were used to arrange, and present observed results, and the tests were predominantly of a descriptive nature. Untransformed pH and log_10_ transformed CFU/mL data were used for statistical and inferential analyses. The descriptive statistics were mean and standard deviation. In terms of inferential statistics, two-way and three-way ANOVA (*p <* 0.05) followed by Tukey Honest Significant Differences (HSD) post hoc tests were used to determine significant differences (0.0001, 0.001, 0.01 and *p <* 0.05) and arrange differentiated groups, respectively. All statistical analyses and plots were performed in R (v 3.5.1) using the “dplyr”, “agricolae”, “1me4”, “ggplot2”, “ggpubr”, and “ggsignif” packages.

## 5. Conclusions

This incubation study has shown that VFAs with C_2_–C_6_ chain length (acetic to caproic acids) present at concentrations above 2 g/L have antibacterial effects against *E. faecalis* NCTC 00775, *E. coli* JCM 1649 and *K. pneumoniae* A17 within 48 h. The modulators of these effects on the tested strains were VFA concentration, molecular weight, and contact time. The VFA cocktail was the most effective against all the tested strains, having a lower MBC and achieving a 7 log_10_ decrease in bacterial counts within the first 24 h of incubation. This is a good indicator that the naturally obtainable combination of the VFAs in AD has the potential as an antibacterial agent. *E. faecalis* was the most resistant strain, being eliminated by VFAs mostly at the MBC of 4 g/L and not being susceptible to low molecular weight VFAs, while *K. pneumoniae* was the most susceptible strain with the VFAs MBC of 2 g/L. It appears that MBC may be a more useful indicator of the antibacterial efficacy of VFAs in AD rather than LC_50_ because the MBC achieves a more significant kill (99.9%), which is more important for waste disinfection than LC_50_ (50% kill). The VFA concentrations that were effective against the bacterial strains lie within the range that can be produced during the acidogenic phase of anaerobic digestion and, hence, provide a further understanding of the sanitary efficacy of AD.

## Figures and Tables

**Table 1:** Percentage decrease in *E. faecalis* NCTC 00775 counts (CFU/mL) at 1, 2 and 4 g/L of VFAs during 48 h mesophilic anaerobic incubation.

Incubation Time (h)	VFA Type	VFA Concentration (g/L)		
		1	2	4
3	Acetic	24.93 (32.91)	25.11 (4.91)	76.71 (7.18)
	Propionic	14.91 (18.23)	40.86 (20.83)	53.80 (7.27)
	Butyric	11.18 (20.25)	33.48 (4.90)	94.94 (0.81)
	Valeric	−135.01	61.57 (7.41)	94.99 (4.43)
	Caproic	−101.60	62.14 (5.74)	99.94 (0.06)
	VFA cocktail	38.17 (53.63)	60.21 (5.47)	94.82 (7.92)
6	Acetic	58.33 (10.75)	57.90 (8.82)	95.85 (0.31)
	Propionic	6.10 (14.12)	47.25 (14.59)	75.36 (10.38)
	Butyric	−25.97	60.83 (1.11)	98.23 (0.47)
	Valeric	−510.02	−105.39	98.71 (0.58)
	Caproic	−297.94	67.22 (13.27)	87.09 (18.89)
	VFA cocktail	1.68 (8.21)	73.44 (3.37)	99.77 (0.23)
24	Acetic	59.79 (7.64)	55.29 (8.69)	98.75 (1.67)
	Propionic	−58.93	50.46 (9.10)	97.42 (1.36)
	Butyric	−187.50	74.60 (2.52)	99.65 (0.12)
	Valeric	−281.02	50.68 (41.83)	100
	Caproic	−176.53	46.32 (41.56)	91.13 (15.34)
	VFA cocktail	−192.38	75.51 (2.33)	100
48	Acetic	76.71 (7.46)	84.85 (3.38)	99.96 (0.01)
	Propionic	−50.09	77.55 (9.74)	99.71 (0.06)
	Butyric	−25.45	78.51 (5.42)	99.96 (0.01)
	Valeric	−199.88	85.21 (5.37)	100
	Caproic	−172.17	76.04 (35.10)	100
	VFA cocktail	−74.89	85.63 (3.73)	100

**Table 2:** Percentage decrease in *E. coli* JCM 1649 counts (CFU/mL) at 1, 2 and 4 g/L of VFAs during 48 h mesophilic anaerobic incubation.

Incubation Time (h)	VFA Type	VFA Concentration (g/L)		
		1	2	4
3	Acetic	−2.33	43.08 (21.43)	73.00 (29.55)
	Propionic	−16.05	−78.98	0.00
	Butyric	−24.61	39.65 (50.06)	31.68 (38.12)
	Valeric	−106.60	−22.47	83.82 (3.09)
	Caproic	−657.08	−89.79	100
	VFA cocktail	−588.17	50.27 (18.64)	100
6	Acetic	−7.82	60.84 (7.24)	99.96 (0.05)
	Propionic	−91.40	−11.81	84.59 (6.29)
	Butyric	−804.28	75.22 (19.40)	92.80 (3.53)
	Valeric	−1839.00	−45.09	100
	Caproic	−2632.90	53.51 (22.01)	100
	VFA cocktail	−5650.18	81.29 (8.09)	100
24	Acetic	−45.12	91.11 (4.19)	100
	Propionic	−9273.02	69.82 (9.75)	100
	Butyric	−8295.16	87.50 (9.25)	100
	Valeric	−3867.83	50.44 (31.53)	100
	Caproic	−2413.23	88.92 (10.56)	100
	VFA cocktail	−3038.80	12.96 (18.43)	100
48	Acetic	−102.66	99.69 (0.47)	100
	Propionic	−9465.82	90.19 (3.05)	100
	Butyric	−8962.35	98.31 (1.14)	100
	Valeric	−4461.74	89.14 (16.19)	100
	Caproic	−1932.47	97.39 (4.51)	100
	VFA cocktail	−1539.65	100	100

**Table 3:** Percentage decrease in *K. pneumoniae* A17 counts (CFU/mL) at 1, 2 and 4 g/L of VFAs during 48 h mesophilic anaerobic incubation.

Incubation Time (h)	VFA Type	VFA Concentration (g/L)		
		1	2	4
3	Acetic	−8.03	13.00 (41.92)	72.70 (34.68)
	Propionic	36.35 (5.77)	62.77 (1.53)	97.51 (2.46)
	Butyric	66.59 (14.75)	53.90 (14.80)	100
	Valeric	39.53 (3.23)	44.87 (4.42)	100
	Caproic	−2312.32	−683.97	99.81 (0.05)
	VFA cocktail	5.51 (8.71)	50.35 (13.21)	100
6	Acetic	15.01 (8.21)	69.74 (17.10)	98.35 (1.66)
	Propionic	35.83 (8.71)	51.93 (9.45)	99.96 (0.06)
	Butyric	39.55 (17.28)	73.77 (7.01)	100
	Valeric	−44.17	71.52 (3.66)	100
	Caproic	−1603.48	83.87 (9.88)	99.98 (0.03)
	VFA cocktail	−724.57	67.76 (15.16)	100
24	Acetic	−13.80	−19.80	99.42 (0.38)
	Propionic	63.18 (8.97)	88.86 (7.70)	100
	Butyric	−123.88	99.79 (0.17)	100
	Valeric	62.35 (13.31)	99.05 (0.55)	100
	Caproic	−1615.55	100	100
	VFA cocktail	98.36 (0.19)	99.15 (0.67)	100
48	Acetic	97.71 (1.67)	97.24 (2.40)	99.90 (0.08)
	Propionic	−28.32	99.93 (0.12)	100
	Butyric	43.54 (76.74)	100	100
	Valeric	86.08 (9.37)	100	100
	Caproic	−668.53	100	100
	VFA cocktail	64.92 (20.72)	99.90	100

**Table 4:** Overview of the antibacterial effect of VFAs against selected bacterial strains at 4 g/L.

VFA (4 g/L)	Most Effective Against	Least Effective Against
Acetic acid	*E. coli*	*K. pneumoniae* *E. faecalis*
Propionic acid		*E. faecalis* *E. coli* *K. pneumoniae*
Butyric acid	*K. pneumoniae*	*E. faecalis*
Valeric acid	*E. faecalis* *E. coli* *K. pneumoniae*	
Caproic acids	*E. faecalis* *E. coli* *K. pneumoniae*	
VFA cocktail	*E. faecalis* *E. coli* *K. pneumoniae*	

**Table 5:** LD 50 (g/L) and MBC (g/L) of *Enterococcus faecalis* during 48 h mesophilic anaerobic incubation.

Time	3 h		6 h		24 h		48 h	
VFAs	LD 50	MBC	LD 50	MBC	LD 50	MBC	LD 50	MBC
Acetic acid	2.41	>4	1.01	>4	1.07	>4	0.77	4
Propionic acid	3.02	>4	2.56	>4	2.46	>4	2.12	>4
Butyric acid	2.60	>4	2.38	>4	2.20	>4	2.13	4
Valeric acid	2.47	>4	3.24	>4	2.21	4	2.09	4
Caproic acid	2.18	4	2.55	>4	2.56	>4	2.11	4
VFA cocktail	1.35	>4	1.71	>4	2.13	4	2.09	4

**Table 6:** LD 50 (g/L) and MBC (g/L) of *Escherichia coli* over 48 h period during mesophilic anaerobic incubation.

Time	3 h		6 h		24 h		48 h	
VFAs	LD 50	MBC	LD 50	MBC	LD 50	MBC	LD 50	MBC
Acetic acid	2.88	>4	2.20	4	1.94	4	1.90	4
Propionic acid	5.50	>4	4.01	>4	2.15	4	2.06	4
Butyric acid	4.41	>4	2.44	>4	2.07	4	1.94	4
Valeric acid	4.01	>4	2.94	4	2.20	4	2.06	4
Caproic acid	2.94	4	2.20	4	2.07	4	1.99	4
VFA cocktail	2.23	4	2.10	4	2.34	4	1.85	2

**Table 7:** LD 50 (g/L) of *Klebsiella pneumoniae* over 48 h period during mesophilic anaerobic incubation.

Time	3 h		6 h		24 h		48 h	
VFAs	LD 50	MBC	LD 50	MBC	LD 50	MBC	LD 50	MBC
Acetic acid	4.37	>4	1.76	>4	3.63	>4	0.08	4
Propionic acid	1.30	>4	1.37	4	0.92	4	0.10	2
Butyric acid	1.06	4	1.24	4	1.85	4	0.82	2
Valeric acid	1.37	4	2.15	4	0.78	4	0.39	2
Caproic acid	3.04	>4	2.11	4	1.85	2	1.85	2
VFA cocktail	1.71	4	1.85	4	0.09	4	0.65	2

## Data Availability

The data presented in this study are available in the article and in the Supplementary Material.

## Supplementary Materials

The following supporting information can be downloaded at: https://www.mdpi.com/article/10.3390/molecules29091908/s1. Table S1: Inferential statistics to determine the significant difference using one-way ANOVA, two-way ANOVA and Tukey Honest post hoc tests, Table S2: Antibiogram of bacterial strains used for broth incubation, Figure S1: Correlation analysis of surviving population (log CFU/mL) (a measure of VFA toxicity) of *E. faecalis* NCTC 00775, *K. pneumoniae* A17, and *E. coli* JCM 1649 against pH.
