# Supplementary material for: Volatile Fatty Acids Effective as Antibacterial Agents against Three Enteric Bacteria during Mesophilic Anaerobic Incubation
Source: Molecules. 2024 Apr 23;29(9):1908. doi: 10.3390/molecules29091908 (PMC11085169; doi:10.3390/molecules29091908)
Supplement: Supplementary file 1 [file molecules-29-01908-s001.zip › molecules-2949664-supplementary.pdf]

Supplementary Materials

Volatile Fatty Acids Effective as Antibacterial Agents against Three Enteric Bacteria during Mesophilic Anaerobic Incubation

Saanu Victoria Otite [1], Alfonso José Lag-Brotons [2], Lawrence I. Ezemonye [3,4], Alastair D. Martin [2], Roger W. Pickup [5] and Kirk T. Semple [1,*]

[1] Lancaster Environment Centre, Library Avenue, Lancaster University, Lancaster LA1 4YQ, UK; s.obatusin@lancaster.ac.uk

[2] Engineering Department, Gillow Avenue, Lancaster University, Lancaster LA1 4YW, UK

[3] Centre for Global Eco-Innovation Nigeria, University of Benin, Benin City PMB 300313, Nigeria

[4] Vice Chancellor's Office, Igbinedion University Okada, Benin City PMB 0006, Nigeria

[5] Division of Biomedical and Life Sciences, Furness Building, Lancaster University, Lancaster LA1 4YG, UK

* Correspondence: k.semple@lancaster.ac.uk; Tel.: +44-(0)-1524-510554

Table S1. Inferential statistics to determine significant difference using one-way and two-way ANOVA, and Tukey Honest post hoc tests.

		***E. coli* JCM 1649**		***K. pneumoniae* A17**		***E. faecalis* NCTC 00775**	
VFA type	Concentration (g/L)	log10 (CFU/mL)	HSD Tukey Post Hoc	log10 (CFU/mL)	HSD Tukey Post Hoc	log10 (CFU/mL)	HSD Tukey Post Hoc
Acetic acid (C2)	0	8.46	a	8.66	a	8.70	a
	1	6.97	b	6.89	b	7.78	b
	2	6.13	b	6.50	b	7.27	b
	4	3.67	c	4.61	c	6.27	c
Propionic acid (C3)	0	8.46	a	8.66	a	8.70	a
	1	7.55	ab	7.66	ab	8.05	b
	2	6.57	b	6.30	b	7.35	c
	4	4.50	c	3.51	c	6.66	d
Butyric acid (C4)	0	8.46	a	8.66	a	8.70	a
	1	7.55	ab	7.25	a	8.35	a
	2	6.57	b	5.44	b	7.35	b
	4	4.50	c	2.02	c	5.89	c
Valeric acid (C5)	0	8.46	a	8.66	a	8.70	a
	1	7.88	ab	7.03	b	8.58	a
	2	6.52	b	5.79	b	7.67	a
	4	3.28	c	2.37	c	4.67	b
Caproic acid (C6)	0	8.46	a	8.66	a	8.70	a
	1	8.01	a	8.60	a	8.55	a
	2	5.56	b	5.09	b	7.43	a
	4	2.39	c	3.16	b	4.10	b
VFA	0	8.46	a	8.66	a	8.70	a

cocktail	1	7.91	a	7.49	ab	7.87	a
	2	5.42	b	6.08	b	7.42	a
	4	2.42	c	2.24	c	4.31	b

Table S2. Antibiogram of bacterial strains used for antibacterial analysis of VFAs. Gram-positive and Gram-negative antibiotic MAST rings were used for the test.

**Antibiotic**	***E. faecalis* NCTC0075**	***E.* coli JCM 1649**	***K. pneumoniae* A17**
Ampicillin (AP) 10ug	S	S	R
Cephalotin (KF) 5 ug	R	R	R
Collistin Sulphate (CO) 25 ug	R	S	S
Gentamicin (GM) 10ug	R	S	S
Streptomycin (S) 10ug	R	R	R
Sulphatriad (ST) 200ug	R	R	R
Tetracycline (T) 25ug	S	S	S
Cotrimoxazole (TS) 25ug	R	S	S
Chloramphenicol (C) 25 ug	R	S	S
Erythromycin (E) 5 ug	R	R	R
Fusidic acid (FC) 10ug	R	R	R
Oxacillin (OX) 5ug	R	R	R
Novobiocin (NO) 5ug	R	R	R
Penicillin G (PG) 1 unit	R	R	R

a

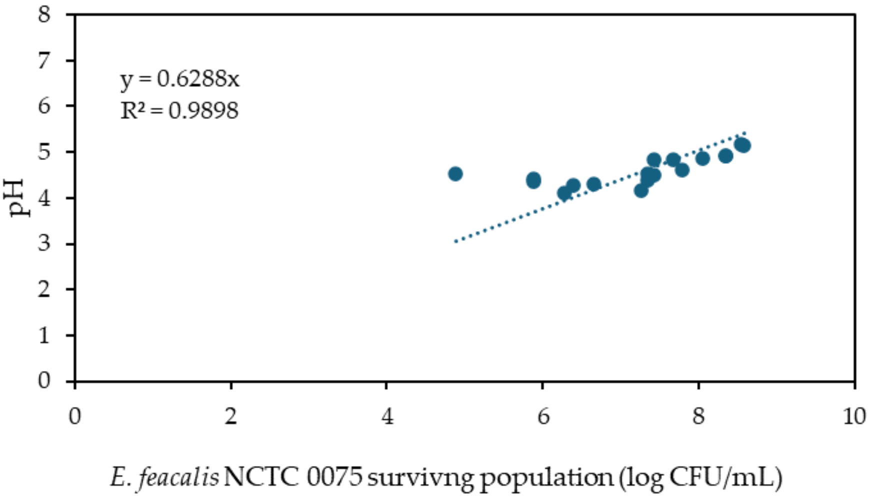

b

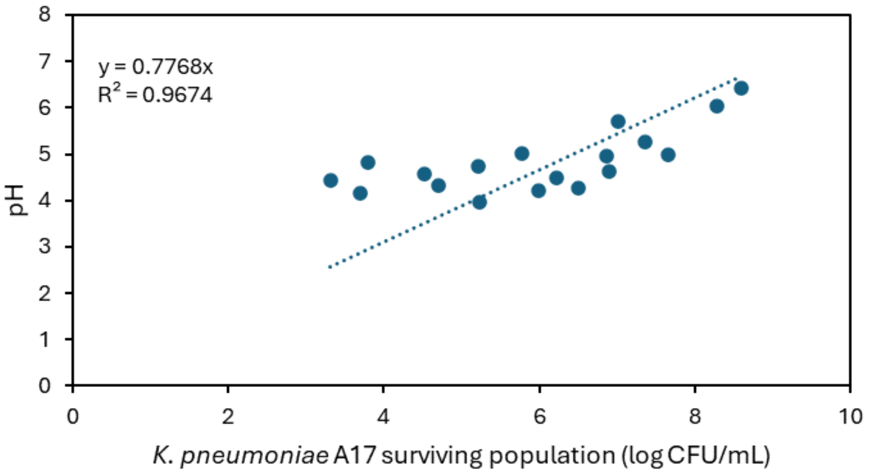

c

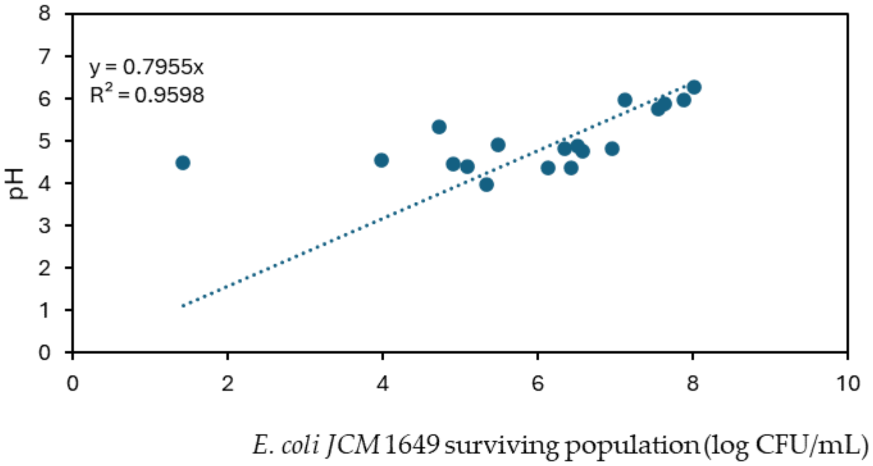

Figure S1. Correlation analysis of surviving population (log CFU/mL) (a measure of VFA toxicity) of *E. faecalis* NCTC 00775 (a) *K. pneumoniae* A17 (b) and *E. coli* JCM 1649 (c) against pH. VFA toxicity correlated positively with pH with R^2 values of 0.9898, 0.9674 and 0.9598 respectively for the three strains.
